# Cell Surface Proteomics Reveals Hypoxia-Regulated Pathways in Cervical and Bladder Cancer

**DOI:** 10.3390/proteomes13030036

**Published:** 2025-08-05

**Authors:** Faris Alanazi, Ammar Sharif, Melissa Kidd, Emma-Jayne Keevill, Vanesa Biolatti, Richard D. Unwin, Peter Hoskin, Ananya Choudhury, Tim A. D. Smith, Conrado G. Quiles

**Affiliations:** 1 Division of Cancer Sciences, School of Medical Sciences, Faculty of Biology, Medicine and Health, The University of Manchester, Manchester M13 9PT, UK; fa.alanazi@qu.edu.sa (F.A.); a.sharif@mu.edu.sa (A.S.); luisa.biolatti@manchester.ac.uk (V.B.); r.unwin@manchester.ac.uk (R.D.U.); peterhoskin@nhs.net (P.H.); ananya.choudhury@nhs.net (A.C.); conrado.guerreroquiles@manchester.ac.uk (C.G.Q.); 2 Department of Radiologic Technology, College of Applied Medical Sciences, Qassim University, Buraydah 51452, Saudi Arabia; 3 Department of Radiological Sciences and Medical Imaging, College of Applied Medical Science, Majmaah University, Al Majmaah 11952, Saudi Arabia; 4 BioMS Core Facility, University of Manchester, Manchester M13 9PT, UK; melissa.kidd@manchester.ac.uk (M.K.); emma.keevill@manchester.ac.uk (E.-J.K.); 5 Mount Vernon Cancer Centre, Northwood M20 4BX, UK; 6 Christie Hospital NHS Foundation Trust, Manchester M20 4BX, UK; 7 Nuclear Futures Institute at Bangor University, Bangor, Gwynedd LL57 2DG, UK

**Keywords:** hypoxia, cell surface biotinylation, plasma membrane proteomics, cervical cancer, bladder cancer, LC-MS/MS

## Abstract

Background Plasma membrane proteins (PMPs) play key roles in cell signalling, adhesion, and trafficking, and are attractive therapeutic targets in cancer due to their surface accessibility. However, their typically low abundance limits detection by conventional proteomic approaches. Methods: To improve PMP detection, we employed a surface proteomics workflow combining cell surface biotinylation and affinity purification prior to LC-MS/MS analysis in cervical (SiHa) and bladder (UMUC3) cancer cell lines cultured under normoxic (21% O_2_) or hypoxic (0.1% O_2_) conditions. Results: In SiHa cells, 43 hypoxia-upregulated proteins were identified exclusively in the biotin-enriched fraction, including ITGB2, ITGA7, AXL, MET, JAG2, and CAV1/CAV2. In UMUC3 cells, 32 unique upregulated PMPs were detected, including CD55, ADGRB1, SLC9A1, NECTIN3, and ACTG1. These proteins were not observed in corresponding whole-cell lysates and are associated with extracellular matrix remodelling, immune modulation, and ion transport. Biotinylation enhanced the detection of membrane-associated pathways such as ECM organisation, integrin signalling, and PI3K–Akt activation. Protein–protein interaction analysis revealed links between membrane receptors and intracellular stress regulators, including mitochondrial proteins. Conclusions: These findings demonstrate that surface biotinylation improves the sensitivity and selectivity of plasma membrane proteomics under hypoxia, revealing hypoxia-responsive proteins and pathways not captured by standard whole-cell analysis.

## 1. Introduction

Plasma membrane proteins (PMPs) are essential for diverse cellular processes, including signal transduction, adhesion, and trafficking. Acting as receptors, transporters, and carriers, they enable cells to respond dynamically to extracellular cues [1]. Owing to their surface accessibility, PMPs are prominent drug targets—comprising nearly two-thirds of all protein-based therapeutics. PMPs are ideal therapeutic targets due to their accessibility, allowing direct interaction with drugs and antibodies [2].

Hypoxia (<2% O_2_), a common feature of solid tumours, is an independent prognostic marker. PMPs mediate signalling and stress responses under hypoxia, and their compositional shifts may offer novel therapeutic targets [3,4]. Hypoxia is known to induce substantial alterations in the cell surface proteome across various in vitro cancer models. These changes often reflect cellular efforts to adapt to metabolic stress and maintain homeostasis under low oxygen conditions. Common hypoxia-responsive surface proteins include integrins, transporters such as glucose transporter 1 (GLUT1), and receptor tyrosine kinases, which facilitate enhanced nutrient uptake, altered adhesion, and survival signalling [5,6,7]. In breast, lung, and pancreatic cancers, hypoxia has been shown to promote extracellular matrix (ECM) remodelling, contributing to tumour progression [8]. Understanding these hypoxia-induced modifications is crucial for interpreting disease-relevant processes and identifying potential therapeutic targets.

The therapeutic relevance of PMPs is exemplified by approved strategies in oncology. For instance, the sodium-iodide symporter in thyroid cancer enables selective radioactive iodine uptake. In prostate cancer, prostate-specific membrane antigen (PSMA) has been successfully targeted with [^177^Lu-PSMA-617], improving survival in metastatic cases (ClinicalTrials.gov Identifier: NCT03511664). Similarly, somatostatin receptors in pancreatic neuroendocrine tumours are exploited for radiolabelled peptide therapy, as shown in the NETTER-1 trial [9]. In clear cell renal cell carcinoma (ccRCC), the hypoxia-inducible PMP carbonic anhydrase IX (CA9) has been targeted with [^177^Lu-DOTA-hG250], enhancing immunotherapy efficacy and tumour selectivity [10]. These cases highlight the translational potential of PMPs in molecular imaging, therapy, and theranostics.

Although about 30% of genes encode PMPs, these proteins are often underrepresented in proteomic studies, impairing the discovery of novel therapeutic targets [2,11]. This underrepresentation is primarily attributed to the relatively low abundance of PMPs compared to other cellular compartments (e.g., cytoskeleton), which presents a technical challenge for non-targeted mass spectrometry (MS) discovery approaches. To overcome this issue, enrichment techniques, such as ultracentrifugation and cell surface biotinylation, have been developed to isolate PMPs [12,13]. However, ultracentrifugation lacks specificity, as it separates organelles based on size, shape, and density rather than function or location [14,15]. In contrast, cell surface biotinylation specifically targets PMPs, allowing for higher specificity [16]. This last method reduces interference from high-abundance intracellular proteins, enhancing sensitivity and selectivity for PMPs enabling more target discovery [17,18].

We evaluated a cell-surface biotinylation pipeline to characterise hypoxia-induced changes in the PMP landscape of cervical and bladder cancer cells—two tumours influenced by hypoxia-driven progression, therapeutic resistance, and poor prognosis. In cervical cancer, hypoxia is a well-established marker of treatment failure, with 30–50% of patients experiencing recurrence within two years of chemoradiotherapy [19,20]. Muscle-invasive bladder cancer (MIBC) also exhibits of tumour hypoxia—reported in over 70% of cases—which contributes to poor response to chemotherapy. Recurrence rates in MIBC can reach up to 50% within three years post-cystectomy [21,22].

## 2. Materials and Methods

### 2.1. Cell Culture

UMUC3 (bladder) and SiHa (cervical) cancer cell lines, obtained from the American Type Culture Collection (ATCC), were cultured in Eagle’s Minimum Essential Medium (EMEM) and Dulbecco’s Modified Eagle Medium (DMEM), respectively. All media were supplemented with 10% FBS and 2 mM L-glutamine. Cells were routinely maintained in an incubator (LEEC Limited, Nottingham, UK) at 37 °C with 5% CO_2_ up to a maximum confluency of 90%. Cells were exposed to hypoxia (0.1% O_2_) for 48 h using a Whitley H35 Hypoxystation (Don Whitley Scientific, Bingley, West Yorkshire, UK). Control cells were maintained alongside in normoxia (21% O). A total of 18 biological replicates were prepared for SiHa cells (9 hypoxia, 9 normoxia) and 24 for UMUC3 cells (12 hypoxia, 12 normoxia). The replicates were generated across multiple passages, with one set per condition cultured on the same day and the remaining sets on different days to ensure reproducibility, capture biological variability and support robust statistical analysis.

### 2.2. Biotinylation and Isolation of PMPs

Sulpho-NHS-SS-biotin, lysis buffer, NeutrAvidin agarose, wash buffer, column accessory pack, and dithiothreitol (DTT) used for biotinylation and isolation of PMPs were obtained from the Pierce™ Cell Surface Protein Biotinylation and Isolation Kit (ThermoFisher Scientific, Waltham, MA, USA). Biotinylation and isolation of PMPs was performed following the manufacturer’s protocol as described below. Importantly, Sulpho-NHS-SS-Biotin is a membrane-impermeable reagent that selectively reacts with primary amines on surface-exposed lysine residues. Its hydrophilic sulphonate group prevents penetration through the plasma membrane, thereby enabling specific labelling of extracellular-facing plasma membrane proteins and minimising intracellular protein contamination.

Cells were washed twice with Dulbecco’s Phosphate-Buffered Saline (DPBS; Sigma-Aldrich, Gillingham, UK), and incubated with sulpho-NHS-SS-biotin (0.56 mM in DPBS; 10 min, 37 °C). After incubation, the biotin solution was removed, and cells were washed twice with ice-cold Tris-Buffered Saline (TBS; 25 mM Tris, 150 mM NaCl, pH 7.2). Cells were then scraped, rinsed twice with washing buffer, and centrifuged at 500× *g* for 5 min at 4 °C. Supernatant was discarded.

The resulting cell pellet was resuspended in lysis buffer containing 1× protease inhibitors (Cell Signaling Technology Europe B.V., London, UK) by pipetting. The lysate was transferred to 1.5 mL Bioruptor Plus TPX Microtubes (Diagenode, Liege, Belgium) and sonicated using a Diagenode Bioruptor Sonicator (Diagenode, Liege, Belgium) for five cycles of 10 s on and 30 s off at 4 °C. The lysate was then centrifuged at 15,000× *g* for 15 min at 4 °C, and the supernatant was collected in low-protein-binding Eppendorf tubes (Eppendorf, Hamburg, Germany).

Cell lysates were then quantified using the Pierce™ BCA Protein Assay Kit (ThermoFisher Scientific, Waltham, MA, USA) according to the manufacturer’s instructions. A total of 200 µg of protein was added to a column pre-treated with NeutrAvidin agarose and incubated at room temperature for 30 min with end-over-end mixing. After incubation, columns were centrifuged at 1000× *g* for 1 min at room temperature and washed four times with washing buffer. Column was then washed three times with 100 mM triethylammonium bicarbonate (TEAB; ThermoFisher Scientific, Lengnau, Switzerland), and centrifuged at 1000× *g* for 1 min at room temperature.

Captured biotinylated proteins were eluted from the column by incubation with lysis buffer containing 10 mM DTT for 45 min with end-over-end mixing on a rotator. The column was then transferred to a low-protein binding Eppendorf collection tube (Eppendorf), centrifuged at 2000× *g* for 1 min at room temperature, and the flow-through containing the isolated proteins was collected for further downstream liquid chromatography–tandem mass spectrometry (LC-MS/MS) analysis.

### 2.3. Sample Preparation for MS Downstream Analysis

Sample preparation for downstream MS analysis utilised alkylation solution, Trypsin/Lys-C Protease Mix (MS Grade), digestion stop solution, peptide clean-up columns, wash solution A, wash solution B, and elution solution, all provided in the EasyPep™ Mini MS Sample Prep Kit (ThermoFisher Scientific, Waltham, MA, USA). MS samples preparation was performed following the manufacturer’s protocol as described below.

Proteins were incubated with 50 µL of alkylation solution for 30 min at 37 °C. Following alkylation, proteins were digested with 50 µL of Trypsin/Lys-C Protease Mix, and incubated for 3 h at 37 °C on an Orbital incubator SI50 shaker (Jencons-PLS, East Grinstead, UK) at 150 revolution/min. Digestion reaction was stopped by adding 50 µL of digestion stop solution.

After digestion, 300 µL of the sample was transferred to a peptide clean-up column and subjected to sequential washing steps: once with 300 µL of wash buffer A and twice with 300 µL of wash buffer B. Each washing step was followed by centrifugation at 1000× *g* for 1 min at room temperature.

Peptides were eluted by adding 300 µL of elution buffer and centrifuging at 2000× *g*, for 2 min at room temperature. Elution was collected in low-protein binding Eppendorf tubes (Eppendorf). Eluted peptides were lyophilised using Eppendorf Vacuum Concentrator 5301 (Eppendorf).

### 2.4. Whole-Cell Lysate Processing

Whole-cell lysates were prepared using the same protocol as the biotin-enriched samples, excluding biotin labelling, NeutrAvidin binding, and associated wash steps. Experimental condition, cell lysis, protein quantification, digestion, peptide clean-up, and LC-MS/MS preparation were performed identically to the biotin-enriched fraction.

### 2.5. LC-MS/MS Analytical Methodology

#### 2.5.1. Chromatographic Separation (Loop-Out Injection)

Peptides were resuspended in 0.1% formic acid in water. The separation was conducted using a Thermo RSLC system equipped with an NCP3200RS nano pump, WPS3000TPS autosampler, and TCC3000RS column oven (ThermoFisher Scientific, Waltham, MA, USA). Buffer A consisted of 0.1% formic acid in water, and buffer B consisted of 0.1% formic acid in acetonitrile. A sample injection volume of 2 µL was loaded into the end of a 5 µL loop and reverse-flushed onto the analytical column (Waters nanoEase™ M/Z Peptide CSH C18 Column, 130 Å, 1.7 µm, 100 µm × 100 mm), maintained at 35 °C. The flow rate was set to 300 nL/min for 8 min, with an initial pulse of 500 nL/min for 0.3 min to rapidly pressurise the column. The injection valve was set to load mode before initiating a multistage gradient separation. The gradient began with 1% buffer B, which increased to 6% over 2 min, followed by a gradual increase to 18% over 44 min. The percentage of buffer B was further increased to 29% over 7 min, then to 65% over 1 min. The column was subsequently washed for 4 min at 65% buffer B, followed by a return to 2% buffer B within 1 min. The total run time for the method was 75 min.

#### 2.5.2. MS Source Parameters

The analytical column was coupled to a Thermo Exploris™ 480 mass spectrometer via a Thermo nanospray Flex Ion Source using a 20 µm ID fused silica capillary (ThermoFisher Scientific, Waltham, MA, USA). Capillary was connected to a stainless-steel emitter (outer diameter 150 µm, inner diameter 30 µm; Thermo Scientific, ES542) via a butt-to-butt connection in a steel union. Nanospray voltage was set to 1900 V, and the ion transfer tube temperature was maintained at 275 °C.

#### 2.5.3. MS/MS Acquisition Settings

Data acquisition was performed in a data-dependent manner with a fixed cycle time of 1.5 s and an expected peak width of 15 s. Full MS data were acquired in positive ion mode over a scan range of 300–1750 Th with a resolution of 120,000, a normalised AGC target of 300%, and a maximum fill time of 25 ms for a single microscan. Fragmentation data were acquired from precursor ions with charge states of +2 or +3 and an intensity threshold greater than 5000. Precursors were dynamically excluded from further analysis for 15 s within a 10 ppm window following a single acquisition. Fragmentation spectra were recorded at a resolution of 15,000, with a normalised collision energy of 30%, a normalised AGC target of 300%, a first mass of 110 Th, and a maximum fill time of 25 ms for a single microscan. All data were collected in profile mode to ensure high-resolution analysis suitable for downstream interpretation.

#### 2.5.4. Protein Identification and Quantitative Analysis

Raw files were analysed using Proteome Discoverer (v3.1, ThermoFisher Scientific, Waltham, MA, USA) with the integrated CHIMERYS™ search engine (v3.0.0, MSAID, Munich, Germany). Workflow configurations were as provided by the BioMS Core Facility at the University of Manchester. The CHIMERYS engine replaced the Sequest HT and Percolator nodes, alongside a Top N Peaks filter. Peptide identification was performed against the SwissProt human reference proteome (TaxID 9606; release 10 February 2024) using strict trypsin specificity (cleavage at lysine and arginine, except before proline), allowing a maximum of two missed cleavages. Searches included precursor ions with charge states from +1 to +6, with carbamidomethylation of cysteine (+57.021 Da) as a fixed modification and oxidation of methionine (+15.995 Da) as a variable modification. The reference database primarily contains canonical sequences and does not comprehensively include alternative splice variants or post-translationally modified proteoforms. As such, protein identifications represent canonical forms and do not reflect proteoform-level resolution. A precursor mass tolerance of 10 ppm and fragment mass tolerance of 0.02 Da were appTlied. Raw proteomic data were normalised based on the total number of peptides per sample, ensuring comparability across runs. Scaling was applied to the average values across biological replicates to further minimise technical variation between conditions. Protein and peptide false discovery rates (FDRs) were estimated using a target-decoy strategy, and results were filtered to retain identifications at a strict FDR of 1%, with high-confidence proteins reported at a protein-level FDR threshold of 5%. Proteomic analyses were conducted at the University of Manchester BioMS Core Facility (RRID: SCR_020987).

### 2.6. In Silico Proteomic Data Analysis

Normalised proteomic data were analysed using R Studio (version 2024.04.2) with the tidyverse, dplyr, and qvalue package packages to identify, filter missing values, and calculate the false discovery rate (FDR) across biological replicates. Only proteins detected in at least 30% of the samples were retained for downstream analysis.

For the SiHa dataset, FDR values were calculated using Storey’s q-value method. Proteins with FDR < 0.05 and an absolute log_2_ fold change (|log_2_FC| ≥ 1) were considered significantly differentially abundant. This approach was selected due to the lower variability and moderate replicate size in the SiHa dataset, making q-value estimation more suitable for identifying a high-confidence set of candidates while maintaining strict FDR control.

In contrast, for the UMUC3 dataset, which comprised more replicates (*n* = 24) and exhibited higher variability, statistical significance was assessed using Benjamini–Hochberg (BH) adjusted *p*-values < 0.05, alongside the same |log_2_FC| threshold. The BH method was chosen to enhance sensitivity for downstream enrichment analyses (e.g., Gene Ontology, Reactome), without compromising the control of false positives. Both q-value and BH are established FDR-controlling procedures, and their use was tailored to the structure and statistical properties of each dataset. To address potential concerns, we provide a supplementary comparison of UMUC3 results filtered using q-value-based FDR estimates versus BH-adjusted *p*-values (Supplementary Figure S1), highlighting differences in the number and identity of differentially abundant proteins. To ensure transparency and reproducibility, we include a supplementary comparison of UMUC3 results filtered using both q-values (FDR-based) and Benjamini–Hochberg (BH) adjusted *p*-values (Figure S1). Functional enrichment analysis of differentially abundant proteins was performed using the STRING database https://string-db.org (accessed on 15 June 2025). 

## 3. Results and Discussion

### 3.1. Protein Detection in Biotin-Based vs. Whole-Cell Fractions

**Figure 1:**
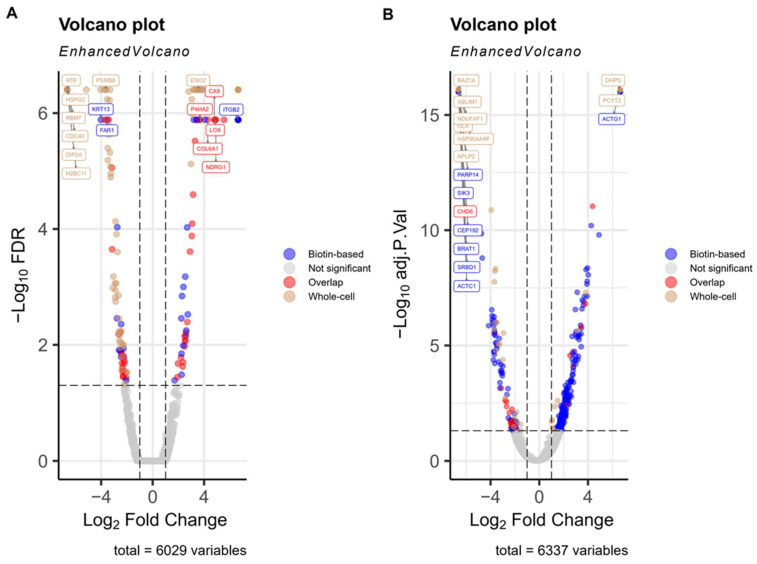
Volcano plots of differentially abundant proteins under hypoxia in SiHa and UMUC3 cells. (**A**) SiHa cervical cancer cells and (**B**) UMUC3 bladder cancer cells were cultured under hypoxic conditions (0.1% O_2_) for 48 h. Plasma membrane proteins were isolated using biotin-based surface enrichment, while total cellular proteins were obtained via whole-cell lysis. Following LC-MS/MS analysis, differential protein abundance was determined by comparing hypoxia vs. normoxia, with significance thresholds set at FDR < 0.05 and |log_2_ fold change| ≥ 1. Each volcano plot displays log_2_ fold change (*x*-axis) versus –log_10_ FDR (*y*-axis) for all quantified proteins. The total number of proteins shown (6029 for SiHa and 6337 for UMUC3) represents the combined set of proteins identified in either the biotin-enriched fraction, the whole-cell lysate fraction, or both for each cell line. Proteins detected in both fractions were counted once, and proteins found in only one fraction were also included, reflecting the full set of proteins evaluated for differential abundance.

To compare overall proteomic coverage between biotin-based surface enrichment and whole-cell lysate approaches, we evaluated the total number of proteins identified by LC-MS/MS in each method across both SiHa and UMUC3 cell lines. In SiHa cells, 4792 proteins were identified in the biotin-enriched fraction, while 5128 proteins were detected in whole-cell lysates. Similarly, in UMUC3 cells, 4385 proteins were detected in the enriched fraction compared to 5471 in the whole-cell samples (Figure 1A,B). The total number of proteins evaluated for differential abundance (6029 in SiHa and 6337 in UMUC3) represents the combined set of proteins identified in either the biotin-enriched or whole-cell lysate fractions, including those detected in only one fraction as well as those shared between both.

### 3.2. Hypoxia Induced Change in PMPs Abundance Demonstrated in Biotin-Enriched Fractions

**Figure 2:**
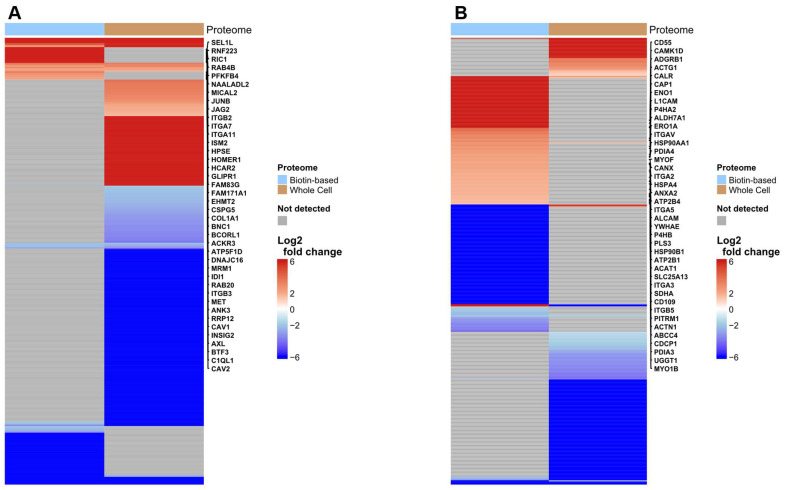
Heatmaps of Differentially abundant proteins in Biotin-Enriched and Whole-Cell Fractions of SiHa and UMUC3 Cells Under Hypoxia. SiHa and UMUC3 cells were cultured under normoxia (21% O_2_) and hypoxia (0.1% O_2_) for 48 h. Both biotin-enriched membrane proteins and whole-cell lysates were analysed. (**A**) SiHa: proteins with |log_2_FC| ≥ 1 and FDR < 0.05. Gene names shown for the top 40 detected in the biotin-enriched fraction only. (**B**) UMUC3: proteins with |log_2_FC| ≥ 1 and adjusted *p* < 0.05. Gene names shown for the top 40 detected in the biotin-enriched fraction only. To avoid overlapping, arrows were added from each gene’s original row; gene names may not align directly with their rows.

The potential of the discovery pipeline to identify PMPs upregulated on hypoxic cells was examined. Significantly differentially abundant proteins detected in the biotin-enriched fractions only of SiHa and UMUC3 cells under hypoxic conditions were determined (Figure 2).

The biotin-enriched proteome from SiHa cells (Figure 2A) included proteins associated with cell–matrix interaction, receptor signalling, and extracellular remodelling. Notably, integrin subunits (ITGA7, ITGA11, ITGB2, ITGB3) and caveolar proteins (CAV1, CAV2) were among the most highly upregulated, consistent with plasma membrane localisation and roles in adhesion and signal transduction [23,24]. Receptors such as hepatocyte growth factor receptor (MET), receptor tyrosine kinase (AXL), Jagged Canonical Notch Ligand 2 (JAG2), and atypical chemokine receptor 3 (ACKR3) were also identified as hypoxia upregulated in the enriched dataset. These proteins are known to mediate cellular responses to extracellular cues and are often regulated by hypoxic signalling pathways in various tumour contexts [25]. The absence of these proteins from the whole-cell fraction suggests that surface biotinylation improves sensitivity for membrane-tethered receptors that may otherwise be underrepresented in total lysates due to low abundance.

A similar analysis of UMUC3 cells (Figure 2B) under hypoxia revealed a distinct but equally informative surface profile, with prominent enrichment of plasma membrane proteins. Among the highly upregulated candidates was Complement Decay-Accelerating Factor (CD55), a GPI-anchored complement-regulatory protein known to promote tumour immune evasion by inhibiting complement-mediated lysis [26,27]. Other upregulated PMPs included Adhesion G Protein-Coupled Receptor B1 (ADGRB1), implicated in phagocytosis and immune signalling [28], Calcium/Calmodulin-Dependent Protein Kinase 1D (CAMK1D), a stress-responsive kinase known to contribute to tumour immune resistance [29], and Calmodulin-Like Protein 5 (CALML5), a calcium-binding protein localised to the plasma membrane in differentiating epithelial cells [30]. Cytoskeletal proteins such as and Actin Alpha Cardiac Muscle 1 (ACTC1), which are involved in membrane organisation, actin filament remodelling, and cell motility, were also upregulated in the enriched proteome [31,32,33].

In addition to these plasma membrane components, several nuclear and mitochondrial proteins were upregulated within the biotinylated membrane proteome. Although these are traditionally associated with intracellular compartments, emerging evidence suggests that certain nuclear and mitochondrial proteins may relocate to the cell surface under stress or in malignancy [34]. One such example in our dataset is calcium/calmodulin-dependent protein kinase 1D (CAMK1D), a kinase known to be upregulated under hypoxic stress and implicated in calcium signalling and tumour progression [29]. Similarly, Actin Gamma 1 (ACTG1), a key cytoskeletal protein involved in actin filament organisation, plays a critical role in cancer cell motility and invasion [32,33], and its detection at the surface may reflect cytoskeletal reorganisation under hypoxic stress. ATP synthase F1 subunit delta (ATP5F1D), a mitochondrial ATP synthase subunit, was also detected in the membrane-enriched fraction; under hypoxic stress, mitochondrial dysfunction and altered dynamics may lead to the mis-localisation of mitochondrial proteins like ATP5F1D to the plasma membrane [35]. The presence of such proteins highlights the complexity of the hypoxic surface proteome and underscores the importance of complementary validation strategies for precise localisation [36,37].

### 3.3. Biotin-Baed Enricment Revels Additonal Hypoxia-Related Changes in Plasma Membrane-Associated Functional Pathways

To assess the biological process and subcellular distribution of proteins identified using biotin-enriched and whole-cell proteomics, we performed enrichment analyses of differentially abundant proteins in both SiHa and UMUC3 cell lines. Gene Ontology (GO) terms for Cellular Component (CC) and Biological Process (BP), as well as pathway annotations from Reactome, and KEGG were used to evaluate the compartment specificity and functional roles of the proteomes. This approach enabled a deeper understanding of how biotin-based surface labelling influences the detection of differentially abundant membrane-associated proteins and context-specific signalling pathways under hypoxic stress.

**Figure 3:**
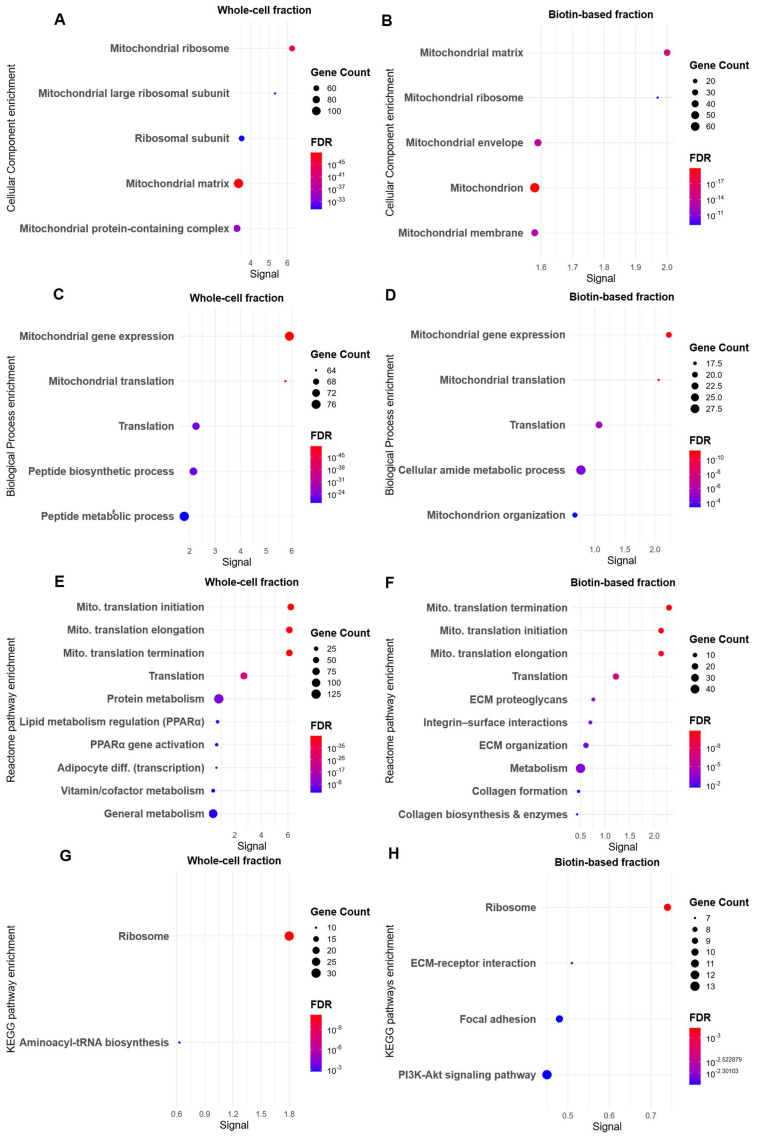
GO and Pathways Enrichment Analyses of Differentially abundant proteins in SiHa Cervical Cancer Cells Under Hypoxia. Differentially abundant proteins (log_2_FC| ≥ 1, FDR < 0.05) were identified in SiHa cells cultured under normoxia (21% O_2_) and hypoxia (0.1% O_2_) for 48 h, using either a biotin-based plasma membrane enrichment method or conventional whole-cell lysate extraction. Enrichment analyses were conducted using STRING (v12). (**A,B**) GO Cellular Component (CC) enrichment. (**A**) Whole-cell lysate; (**B**) Biotin-based fraction. (**C,D**) GO Biological Process (BP) enrichment. (**C**) Whole-cell lysate; (**D**) Biotin-enriched fraction. (**E,F**) Reactome pathway enrichment. (**E**) Whole-cell lysate; (**F**) Biotin-based fraction. (**G,H**) KEGG pathway enrichment. (**G**) Whole-cell lysate; (**H**) Biotin-based fraction. Signal represents the pathway enrichment score, which reflects the strength of enrichment based on the number of mapped proteins and their statistical significance. Abbreviations: Mito, mitochondrial; diff, differentiation.

CC and BP enrichment revealed distinct patterns between the two cell lines. GO enrichment analysis in SiHa cells revealed minimal distinction between the biotin-enriched and whole-cell datasets. CC analysis (Figure 3A,B) unexpectedly highlighted mitochondrial matrix, mitochondrial ribosome, and mitochondrial envelope—compartments not typically associated with biotin-accessible, extracellular domains.

Although mitochondria appeared as a top-enriched term in the CC analysis of the biotin-enriched SiHa fraction, closer examination of the protein list revealed lower abundance and more limited coverage compared to the whole-cell dataset (Table S1). For instance, in the mitochondrial matrix category, the biotin-enriched fraction included 35 proteins. A similar trend was observed for the mitochondrial membrane, where shared proteins such as VDAC1, mitochondrial ribosomal protein S12 (MRPS12), and death-associated protein 3 (DAP3) appeared in both fractions, but the whole-cell dataset showed broader inclusion of key membrane-associated proteins like translocase of outer mitochondrial membrane 5 (TOMM5), translocase of inner mitochondrial membrane 22 (TIMM22), cytochrome c oxidase assembly factor COX16 (COX16), and SRA stem-loop interacting RNA binding protein (SLIRP). This difference suggests that although mitochondrial compartments were detected in the biotin-enriched proteome, they were captured with lower coverage and likely reflect incidental proximity. Notably, mitochondria naturally contain biotin as a coenzyme for several carboxylases, which may contribute to background labelling and explain the low-level mitochondrial presence observed [38].

Similarly, BP analysis (Figure 3C,D) showed enrichment category of mitochondrial gene expression, translation, and amide metabolic processes in both fractions. However, the biotin-enriched fraction contained only 20 proteins involved in mitochondrial gene expression compared to over 60 proteins in the whole-cell fraction, including additional ribosomal subunits, elongation factors, and mitochondrial RNA-binding proteins (Table S1). This consistent reduction in both component- and function-related proteins in the biotin-enriched sample supports the conclusion that the mitochondrial GO signals observed are substantially weaker and less comprehensive, further suggesting that they do not reflect meaningful mitochondrial enrichment.

Reactome analysis of the whole-cell lysate (Figure 3F) showed broad metabolic and transcriptional processes, such as PPARα activation and lipid metabolism [39]. In contrast, the biotin-based fraction (Figure 3E) revealed robust enrichment of plasma membrane–associated signalling processes, including ECM organisation, integrin cell surface interactions, and collagen biosynthesis—hallmarks of TME remodelling and cell–matrix communication [40,41]. KEGG analysis of the whole-cell lysate (Figure 3G) was dominated by ribosomal and aminoacyl-tRNA biosynthesis pathways, reflecting global translation activity. On the other hand, the KEGG analysis of the biotin-based fraction (Figure 3H) further supported membrane selectivity, identifying ECM–receptor interaction, focal adhesion, and PI3K–Akt signalling—well-characterised hypoxia-responsive, membrane-anchored pathways [36,42].

**Figure 4:**
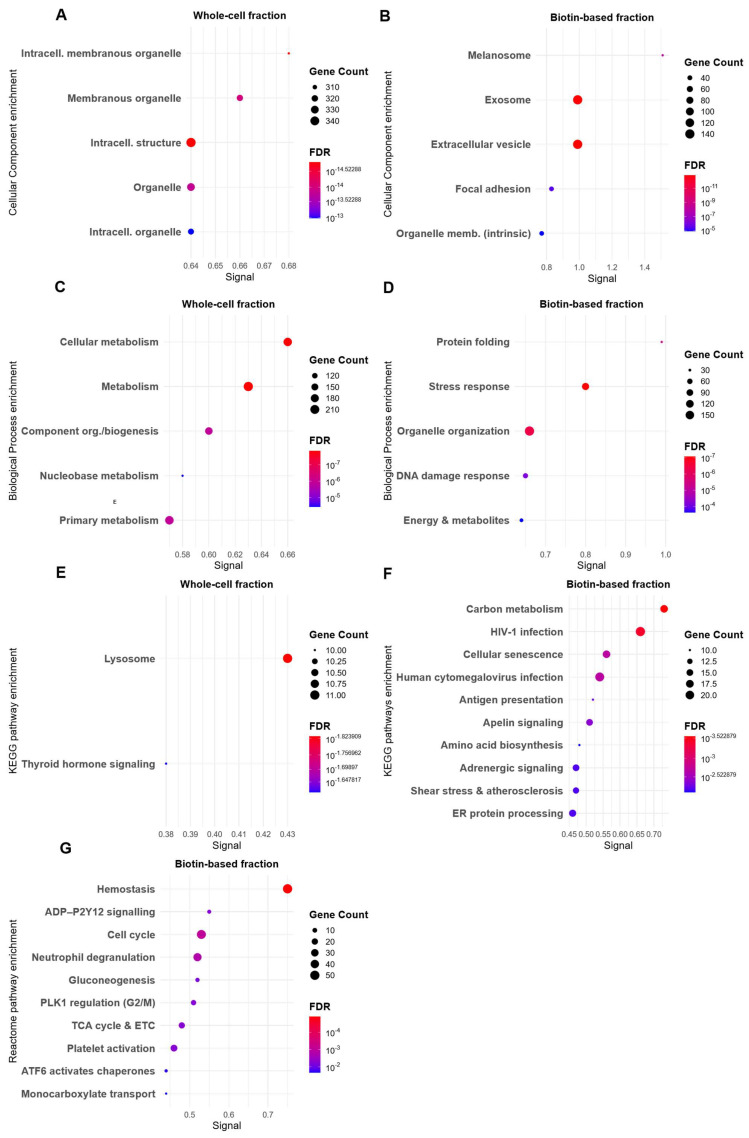
GO and Pathways Enrichment Analyses of Differentially abundant proteins in UMUC3 Bladder Cancer Cells Under Hypoxia. Differentially abundant proteins (|log_2_FC| ≥ 1, adjusted *p*-value < 0.05) were identified in UMUC3 cells cultured under normoxia (21% O_2_) and hypoxia (0.1% O_2_) for 48 h, using either a biotin-based plasma membrane enrichment method or conventional whole-cell lysate extraction. Enrichment analyses were conducted using STRING (v12). (**A**,**B**) GO Cellular Component (CC) enrichment. (**A**) Biotin-enriched fraction; (**B**) Whole-cell lysate. (**C**,**D**) GO Biological Process (BP) enrichment. (**C**) Biotin-enriched fraction; (**D**) Whole-cell lysate. (**E**) Reactome pathway enrichment of the biotin-enriched fraction. (**F**,**G**) KEGG pathway enrichment. (**F**) Biotin-enriched fraction; (**G**) Whole-cell lysate. The Signal represents the pathway enrichment score, which reflects the strength of enrichment based on the number of mapped proteins and their statistical significance. Abbreviations. Mito, mitochondria; intercell, intercellular; memb, membrane; org, organisation; TCA, the citric acid cycle; ECT, electron transport; ER, endoplasmic reticulum.

UMUC3 whole-cell lysate exhibited GO profiles dominated by general intracellular components. CC terms in the whole-cell proteome (Figure 4A) were primarily associated with cytoplasmic and organelle structures, with limited representation of surface-localised compartments. The biotin-based fraction, by comparison, displayed a distinct set of CC terms (Figure 4B), including focal adhesion, extracellular vesicle, extracellular exosome, and extracellular space—features strongly indicative of successful plasma membrane enrichment. A similar contrast was observed in the BP analysis. The whole-cell proteome (Figure 4D) was enriched in fundamental processes such as primary metabolic activity and nucleobase-containing compound metabolism, reflecting a broad intracellular landscape. In contrast, the biotin-enriched proteome (Figure 4C) was characterised by processes related to cellular stress response, protein folding, and organelle organisation—pathways typically associated with membrane signalling and hypoxia-induced adaptation.

Pathway analysis in UMUC3 revealed limited differentiation across enrichment methods. Reactome enrichment (Figure 4G) in the biotin-enriched fraction included broader processes such as hemostasis, neutrophil degranulation, gluconeogenesis, and cell cycle regulation—biologically relevant but not exclusive to membrane signalling. KEGG analysis (Figure 4E,F) showed some membrane-associated enrichment (antigen presentation) in the biotin-labelled proteome, but these were absent from the whole-cell dataset [43].

### 3.4. Protein–Protein Interaction Mapping Reveals ECM–Mitochondrial Crosstalk in Enriched Membrane Fractions

**Figure 5:**
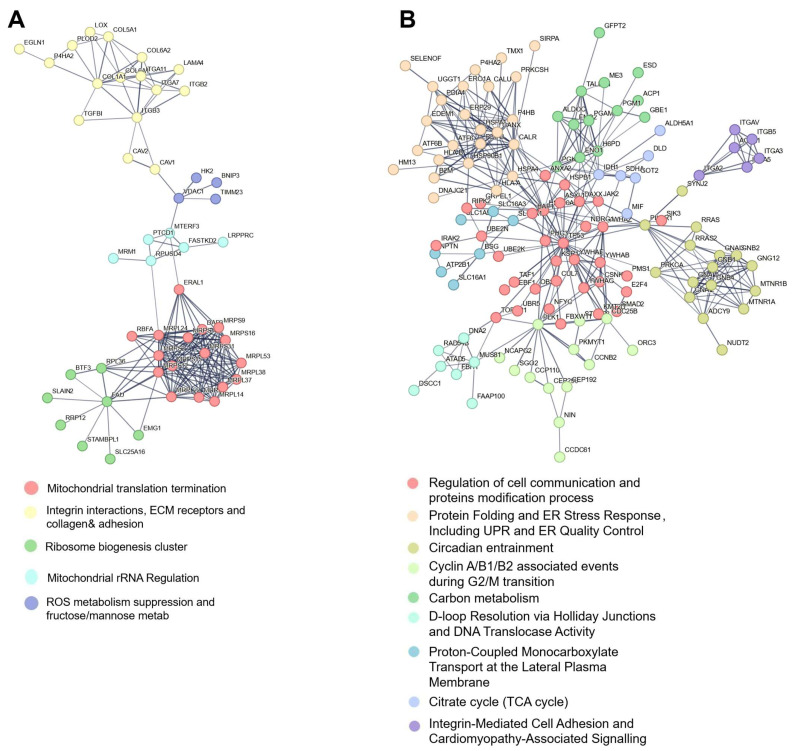
Protein–Protein Interaction (PPI) Networks of Differentially abundant proteins in SiHa and UMUC3 Cancer Cells. (**A**) SiHa biotin-enriched plasma membrane fraction. The network shows differentially abundant proteins (|log_2_FC| ≥ 1, FDR < 0.05) identified from SiHa cells cultured under normoxic (21% O_2_) and hypoxic (0.1% O_2_) conditions for 48 h, following a biotin-based membrane enrichment strategy prior to LC-MS/MS. The resulting network contains 172 nodes and 215 edges, with an average node degree of 2.5 and an average local clustering coefficient of 0.249 (PPI enrichment *p*-value < 1.0 × 10^−16^). (**B**) UMUC3 biotin-enriched plasma membrane fraction. The network shows differentially abundant proteins (|log_2_FC| ≥ 1, FDR < 0.05) from UMUC3 cells cultured under identical conditions and processed using the same enrichment approach. This network comprises 340 nodes and 225 edges, with an average node degree of 1.32 and a clustering coefficient of 0.19 (PPI enrichment *p*-value < 1.0 × 10^−16^). In both panels, nodes represent proteins, and edges represent predicted functional associations. A minimum required interaction scores of 0.7 (high confidence) was applied. Node colours represent distinct functional clusters identified using MCL clustering (inflation = 2). Networks were generated using STRING (v12).

To investigate potential protein–protein interactions between plasma membrane and mitochondrial proteins, we constructed STRING-derived protein–protein interaction (PPI) networks for the biotin-enriched proteomes of SiHa and UMUC3 cells (Figure 5). In the SiHa biotin-enriched network (Figure 5A), ECM-related proteins including COL6A1, Collagen Type VI Alpha 2 Chain (COL6A2), ITGA7, ITGB1, LOX, and Procollagen-Lysine, 2-Oxoglutarate 5-Dioxygenase 2 (PLOD2), all classic markers of ECM remodelling and cell adhesion [40,41], formed a highly connected cluster. Notably, mitochondrial ribosomal proteins (MRPL, MRPS), along with FAST kinase domain-containing protein 2 (FASTKD2) and Era G-protein-like 1 mitochondrial (ERAL1), formed a distinct but adjacent cluster. Bridging proteins such as VDAC1, HK2, and BCL2/adenovirus E1B 19 kDa-interacting protein 3 (BNIP3)—implicated in mitochondrial membrane transport, glycolysis, and mitophagy—served as critical nodes linking ECM-related and mitochondrial protein clusters, suggesting a potential communication axis between the ECM and mitochondria under hypoxic stress. VDAC1 a multifunctional protein in the outer mitochondrial membrane, regulates the exchange of metabolites like ATP and ions, while also modulating apoptosis and cellular metabolism [44,45,46]. BNIP3, a hypoxia-inducible protein, promotes mitophagy to maintain mitochondrial quality under low oxygen conditions, further tying mitochondrial dynamics to stress responses [47]. Cytoskeletal elements, including tubulin and actin, interact with VDAC1 to anchor mitochondria and facilitate their spatial regulation [44,48]. The PPI network analysis suggests an additional layer of ECM–mitochondria crosstalk: VDAC1 associates with CAV1 a caveolae component detected in plasma membrane domains alongside VDAC1. CAV1 is known to interact with integrins, such as ITGB1, to regulate adhesion and signalling. This potential VDAC1–CAV1–integrin axis could link mitochondrial function to ECM cues under hypoxia, consistent with studies showing that caveolae modulate integrin signalling in stiff microenvironments [46,49,50]. Together, these interactions highlight a dynamic interplay between ECM properties and mitochondrial responses, potentially mediated by integrin-associated pathways and cytoskeletal bridges.

In UMUC3, the biotin-enriched network (Figure 5B) displayed a decentralised architecture with multiple functionally distinct hubs. A prominent cluster of solute carriers (SLC) transporters, integrins, and signalling proteins including SRC proto-oncogene, non-receptor tyrosine kinase (SRC), calreticulin (CALR), heat shock protein family A (Hsp70) member 5 (HSPA5), and protein disulfide isomerase family A member 3 (PDIA3) was observed, consistent with active membrane signalling, ER stress response, and protein trafficking (10). Mitochondrial proteins were present but exhibited weaker connectivity to ECM modules than in SiHa. These patterns align with UMUC3′s dominant enrichment of vesicle-mediated signalling, cell adhesion, and glycolytic metabolism [51,52].

The PPI network differences between SiHa and UMUC3 underscore the cell-type-specific relationship between plasma membrane and mitochondrial signalling. In SiHa, the close association between ECM proteins and mitochondrial regulators (HK2, BNIP3, VDAC1) suggests a co-ordinated membrane–organelle stress response, potentially driving metabolic adaptation or apoptosis under hypoxia [53]. In contrast, UMUC3 exhibited a more modular organisation, with distributed responses involving metabolic reprogramming, ER proteostasis, and integrin-mediated signalling.

These data support models in which plasma membrane receptors and adhesion complexes regulate mitochondrial dynamics via mechanical and metabolic signalling [54].

Taken together, our findings demonstrate that biotin-based surface enrichment enhances the detection of plasma membrane-associated proteins and pathways, particularly those involved in cell–ECM interaction, receptor-mediated signalling, and immune modulation. Compared to whole-cell proteomics, this method provided improved coverage of surface-relevant networks, including integrins, receptor tyrosine kinases, and adhesion-related modules. While the degree of enrichment varied between cell lines and annotation platforms, the overall trend of enhanced plasma membrane signal was consistent. Notably, many hypoxia-responsive pathways identified through biotinylation were not detectable in whole-cell lysates, underscoring the added value of targeted surface proteomics for capturing dynamic, low-abundance membrane biology.

Nonetheless, several limitations warrant consideration. The presence of mitochondrial proteins in the biotinylated datasets may reflect either low-level contamination or biologically relevant interactions, such as membrane–mitochondria contact sites under metabolic stress. Hypoxia-induced changes in membrane integrity may also contribute to increased non-specific labelling, despite the use of a non-permeable reagent [54,55]. Notably, mitochondria contain high levels of endogenous biotin, a co-factor for several metabolic enzymes [56]. Therefore, biotin-based enrichment may unintentionally capture mitochondrial fragments or membrane-associated biotinylated enzymes, which in turn could co-isolate interacting partners. Given their high abundance, even limited carryover could result in detectable signals by mass spectrometry. To enhance interpretation of these findings, future studies could incorporate in silico prediction tools, such as SignalP or TMHMM, to identify proteins with signal peptides or transmembrane domains. These bioinformatic filters would strengthen confidence in surface localisation and help distinguish plasma membrane components from intracellular proteins that may appear due to membrane permeabilisation, vesicle fusion, or biotin cofactor presence. Although not applied in the current dataset, such approaches may help refine interpretation of membrane proteomics under stress conditions like hypoxia. Additionally, the reference database primarily contains canonical sequences and does not comprehensively include alternative splice variants or post-translationally modified proteoforms. As such, protein identifications represent canonical forms and do not reflect proteoform-level resolution. This limitation may affect the interpretation of differential abundance and pathway enrichment results, as hypoxia is known to regulate protein stability, function, and localisation through PTMs such as phosphorylation, acetylation, and ubiquitination, which are not captured in this analysis.

To our knowledge, this is the first study to apply cell surface biotinylation-based proteomics under hypoxia in both cervical and bladder cancer models, revealing membrane proteins involved in ECM remodelling, immune evasion, and mitochondrial crosstalk that were undetectable in whole-cell lysates. These findings offer a valuable resource for identifying novel therapeutic targets in hypoxia-adapted tumours. To support the generalisability of our findings, this biotinylation-based enrichment strategy will be applied to additional cervical and muscle-invasive bladder cancer (MIBC) cell lines to validate hypoxia-induced alterations in the plasma membrane proteome. In parallel, orthogonal validation experiments—such as immunofluorescence and Western blotting of membrane fractions—will be applied to confirm the surface localisation of key differentially abundant proteins, including ITGA7 and CD55. These approaches will help strengthen confidence in the spatial specificity of biotin-based enrichment and clarify the biological relevance of proteins with dual or ambiguous subcellular annotation. While biotinylation enhances detection of plasma membrane proteins, it may miss subsets lacking accessible extracellular lysines or localised to lipid rafts. Future studies could combine this approach with complementary methods—such as lectin affinity, gradient fractionation, or metabolic labelling—to broaden coverage and capture additional hypoxia-regulated membrane proteins.

Beyond oncology, cell surface biotinylation could be applied to other hypoxia-associated conditions such as myocardial infarction, stroke, chronic lung disease, and diabetic wounds, where oxygen deprivation modulates plasma membrane signalling involved in inflammation, fibrosis, and tissue repair.

## 4. Conclusions

This study demonstrates that biotin-based proteomic enrichment improves the detection of membrane-associated proteins under hypoxic conditions. Compared to whole-cell lysates, biotin-labelling increased the identification of hypoxia-responsive proteins involved in ECM remodelling, receptor-mediated signalling, and membrane–mitochondrial communication—thereby enhancing the resolution of signalling pathways associated with the hypoxic adaptive response. The comparative analysis across SiHa and UMUC3 cells further revealed cancer-specific patterns of enrichment, highlighting the influence of biological context and proteomic depth. While not without limitations, this approach offers a powerful tool for dissecting spatially organised signalling networks and expands the potential for identifying novel hypoxia-responsive targets in cancer research.

## Data Availability

The data presented in this study are available on request from the corresponding author.

## Supplementary Materials

The following supporting information can be downloaded at: https://www.mdpi.com/article/10.3390/proteomes13030036/s1, Table S1: GO enrichment terms and associated genes identified in SiHa cervical cancer cells; Figure S1: Comparison of Differential Abundant Proteins Results in UMUC3 Cells Using Storey’s q-value vs Benjamini–Hochberg (BH) Adjusted *p*-values UMUC3 bladder cancer cells were cultured under normoxia (21% O_2_) and hypoxia (0.1% O_2_) for 48 h.

## References

[1] Elschenbroich S., Kim Y., Medin J.A., Kislinger T. (2010). Isolation of cell surface proteins for mass spectrometry-based proteomics. Expert Rev. Proteom..

[2] Hopkins A.L., Groom C.R. (2002). The druggable genome. Nat. Rev. Drug Discov..

[3] Vaupel P., Mayer A. (2007). Hypoxia in cancer: Significance and impact on clinical outcome. Cancer Metastasis Rev..

[4] Brown J.M. (2000). Exploiting the hypoxic cancer cell: Mechanisms and therapeutic strategies. Mol. Med. Today.

[5] Hoskin P.J., Sibtain A., Daley F.M., Wilson G.D. (2003). GLUT1 and CAIX as intrinsic markers of hypoxia in bladder cancer: Relationship with vascularity and proliferation as predictors of outcome of ARCON. Br. J. Cancer.

[6] Arriagada C., Silva P., Torres V.A. (2019). Role of glycosylation in hypoxia-driven cell migration and invasion. Cell Adhes. Migr..

[7] Gluck A.A., Aebersold D.M., Zimmer Y., Medova M. (2015). Interplay between receptor tyrosine kinases and hypoxia signaling in cancer. Int. J. Biochem. Cell Biol..

[8] Gilkes D.M., Semenza G.L., Wirtz D. (2014). Hypoxia and the extracellular matrix: Drivers of tumour metastasis. Nat. Rev. Cancer.

[9] Strosberg J., El-Haddad G., Wolin E., Hendifar A., Yao J., Chasen B., Mittra E., Kunz P.L., Kulke M.H., Jacene H. (2017). Phase 3 Trial of (177)Lu-Dotatate for Midgut Neuroendocrine Tumors. N. Engl. J. Med..

[10] Kleinendorst S.C., Oosterwijk E., Molkenboer-Kuenen J., Frielink C., Franssen G.M., Boreel D.F., Tamborino G., Gloudemans M., Hendrikx M., Kroon D. (2024). Towards effective CAIX-targeted radionuclide and checkpoint inhibition combination therapy for advanced clear cell renal cell carcinoma. Theranostics.

[11] Li Y., Qin H., Ye M. (2020). An overview on enrichment methods for cell surface proteome profiling. J. Sep. Sci..

[12] Young J.W., Wason I.S., Zhao Z., Rattray D.G., Foster L.J., Van Hoa F.D. (2020). His-Tagged Peptidiscs Enable Affinity Purification of the Membrane Proteome for Downstream Mass Spectrometry Analysis. J. Proteome Res..

[13] Kuhlmann L., Cummins E., Samudio I., Kislinger T. (2018). Cell-surface proteomics for the identification of novel therapeutic targets in cancer. Expert Rev. Proteom..

[14] Tan S., Tan H.T., Chung M.C. (2008). Membrane proteins and membrane proteomics. Proteomics.

[15] Brown K.A., Tucholski T., Alpert A.J., Eken C., Wesemann L., Kyrvasilis A., Jin S., Ge Y. (2020). Top-Down Proteomics of Endogenous Membrane Proteins Enabled by Cloud Point Enrichment and Multidimensional Liquid Chromatography-Mass Spectrometry. Anal. Chem..

[16] Elia G. (2008). Biotinylation reagents for the study of cell surface proteins. Proteomics.

[17] Liu G., Choi M.H., Ma H., Guo X., Lo P.C., Kim J., Zhang L. (2022). Bioorthogonal Conjugation-Assisted Purification Method for Profiling Cell Surface Proteome. Anal. Chem..

[18] de Boer E., Rodriguez P., Bonte E., Krijgsveld J., Katsantoni E., Heck A., Grosveld F., Strouboulis J. (2003). Efficient biotinylation and single-step purification of tagged transcription factors in mammalian cells and transgenic mice. Proc. Natl. Acad. Sci. USA.

[19] Massobrio R., Bianco L., Campigotto B., Attianese D., Maisto E., Pascotto M., Redda M.G.R., Ferrero A. (2024). New Frontiers in Locally Advanced Cervical Cancer Treatment. J. Clin. Med..

[20] Gennigens C., De Cuypere M., Hermesse J., Kridelka F., Jerusalem G. (2021). Optimal treatment in locally advanced cervical cancer. Expert Rev. Anticancer Ther..

[21] Alfred Witjes J., Max Bruins H., Carrion A., Cathomas R., Comperat E., Efstathiou J.A., Fietkau R., Gakis G., Lorch A., Martini A. (2024). European Association of Urology Guidelines on Muscle-invasive and Metastatic Bladder Cancer: Summary of the 2023 Guidelines. Eur. Urol..

[22] Tian J., Sun J., Fu G., Xu Z., Chen X., Shi Y., Jin B. (2021). Population-based outcome of muscle-invasive bladder cancer following radical cystectomy: Who can benefit from adjuvant chemotherapy?. Transl. Androl. Urol..

[23] Li S., Sampson C., Liu C., Piao H.L., Liu H.X. (2023). Integrin signaling in cancer: Bidirectional mechanisms and therapeutic opportunities. Cell Commun. Signal..

[24] Parton R.G. (2018). Caveolae: Structure, Function, and Relationship to Disease. Annu. Rev. Cell Dev. Biol..

[25] Comoglio P.M., Trusolino L., Boccaccio C. (2018). Known and novel roles of the MET oncogene in cancer: A coherent approach to targeted therapy. Nat. Rev. Cancer.

[26] Bharti R., Dey G., Khan D., Myers A., Huffman O.G., Saygin C., Braley C., Richards E., Sangwan N., Willard B. (2024). Cell surface CD55 traffics to the nucleus leading to cisplatin resistance and stemness by inducing PRC2 and H3K27 trimethylation on chromatin in ovarian cancer. Mol. Cancer.

[27] Pio R., Ajona D., Ortiz-Espinosa S., Mantovani A., Lambris J.D. (2019). Complementing the Cancer-Immunity Cycle. Front. Immunol..

[28] Dyachok J., Shao M.R., Vaughn K., Bowling A., Facette M., Djakovic S., Clark L., Smith L. (2008). Plasma membrane-associated SCAR complex subunits promote cortical F-actin accumulation and normal growth characteristics in Arabidopsis roots. Mol. Plant.

[29] Volpin V., Michels T., Sorrentino A., Menevse A.N., Knoll G., Ditz M., Milenkovic V.M., Chen C.-Y., Rathinasamy A., Griewank K. (2020). CAMK1D Triggers Immune Resistance of Human Tumor Cells Refractory to Anti-PD-L1 Treatment. Cancer Immunol. Res..

[30] Sun B.K., Boxer L.D., Ransohoff J.D., Siprashvili Z., Qu K., Lopez-Pajares V., Hollmig S.T., Khavari P.A. (2015). CALML5 is a ZNF750- and TINCR-induced protein that binds stratifin to regulate epidermal differentiation. Genes Dev..

[31] Lee C.W., Vitriol E.A., Shim S., Wise A.L., Velayutham R.P., Zheng J.Q. (2013). Dynamic localization of G-actin during membrane protrusion in neuronal motility. Curr. Biol..

[32] Suresh R., Diaz R.J. (2021). The remodelling of actin composition as a hallmark of cancer. Transl. Oncol..

[33] Tang Y., Peng X., Huang X., Li J. (2022). Actin gamma 1 is a critical regulator of pancreatic ductal adenocarcinoma. Saudi J. Gastroenterol..

[34] He Q., Li Z. (2021). The dysregulated expression and functional effect of CaMK2 in cancer. Cancer Cell Int..

[35] Wang S., Tan J., Miao Y., Zhang Q. (2022). Mitochondrial Dynamics, Mitophagy, and Mitochondria-Endoplasmic Reticulum Contact Sites Crosstalk Under Hypoxia. Front. Cell Dev. Biol..

[36] Semenza G.L. (2012). Hypoxia-inducible factors: Mediators of cancer progression and targets for cancer therapy. Trends Pharmacol. Sci..

[37] Wheaton W.W., Chandel N.S. (2011). Hypoxia. 2. Hypoxia regulates cellular metabolism. Am. J. Physiol. Cell Physiol..

[38] Tragni V., Primiano G., Tummolo A., Cafferati Beltrame L., La Piana G., Sgobba M.N., Cavalluzzi M.M., Paterno G., Gorgoglione R., Volpicella M. (2022). Personalized Medicine in Mitochondrial Health and Disease: Molecular Basis of Therapeutic Approaches Based on Nutritional Supplements and Their Analogs. Molecules.

[39] Yates J.R., Ruse C.I., Nakorchevsky A. (2009). Proteomics by mass spectrometry: Approaches, advances, and applications. Annu. Rev. Biomed. Eng..

[40] Naba A., Clauser K.R., Ding H., Whittaker C.A., Carr S.A., Hynes R.O. (2016). The extracellular matrix: Tools and insights for the “omics” era. Matrix Biol..

[41] Pickup M.W., Mouw J.K., Weaver V.M. (2014). The extracellular matrix modulates the hallmarks of cancer. EMBO Rep..

[42] Dong P., Konno Y., Watari H., Hosaka M., Noguchi M., Sakuragi N. (2014). The impact of microRNA-mediated PI3K/AKT signaling on epithelial-mesenchymal transition and cancer stemness in endometrial cancer. J. Transl. Med..

[43] Vivanco I., Sawyers C.L. (2002). The phosphatidylinositol 3-Kinase AKT pathway in human cancer. Nat. Rev. Cancer.

[44] Shoshan-Barmatz V., De Pinto V., Zweckstetter M., Raviv Z., Keinan N., Arbel N. (2010). VDAC, a multi-functional mitochondrial protein regulating cell life and death. Mol. Asp. Med..

[45] Maldonado E.N., Lemasters J.J. (2012). Warburg revisited: Regulation of mitochondrial metabolism by voltage-dependent anion channels in cancer cells. J. Pharmacol. Exp. Ther..

[46] Shoshan-Barmatz V., Shteinfer-Kuzmine A., Verma A. (2020). VDAC1 at the Intersection of Cell Metabolism, Apoptosis, and Diseases. Biomolecules.

[47] Zhang J., Ney P.A. (2009). Role of BNIP3 and NIX in cell death, autophagy, and mitophagy. Cell Death Differ..

[48] Rovini A. (2019). Tubulin-VDAC Interaction: Molecular Basis for Mitochondrial Dysfunction in Chemotherapy-Induced Peripheral Neuropathy. Front. Physiol..

[49] Wei Y., Yang X., Liu Q., Wilkins J.A., Chapman H.A. (1999). A role for caveolin and the urokinase receptor in integrin-mediated adhesion and signaling. J. Cell Biol..

[50] Yeh Y.C., Ling J.Y., Chen W.C., Lin H.H., Tang M.J. (2017). Mechanotransduction of matrix stiffness in regulation of focal adhesion size and number: Reciprocal regulation of caveolin-1 and beta1 integrin. Sci. Rep..

[51] Abdelmaksoud N.M., El-Mahdy H.A., Ismail A., Elsakka E.G.E., El-Husseiny A.A., Khidr E.G., Ali E.M., Rashed M.H., El-Demerdash F.E.-S., Doghish A.S. (2023). The role of miRNAs in the pathogenesis and therapeutic resistance of endometrial cancer: A spotlight on the convergence of signaling pathways. Pathol. Res. Pract..

[52] van Niel G., D’Angelo G., Raposo G. (2018). Shedding light on the cell biology of extracellular vesicles. Nat. Rev. Mol. Cell Biol..

[53] Rikka S., Quinsay M.N., Thomas R.L., Kubli D.A., Zhang X., Murphy A.N., Gustafsson A.B. (2011). Bnip3 impairs mitochondrial bioenergetics and stimulates mitochondrial turnover. Cell Death Differ..

[54] Friedman J.R., Nunnari J. (2014). Mitochondrial form and function. Nature.

[55] Habel J.E. (2021). Biotin Proximity Labeling for Protein-Protein Interaction Discovery: The BioID Method. Methods Mol. Biol..

[56] Hollinshead M., Sanderson J., Vaux D.J. (1997). Anti-biotin antibodies offer superior organelle-specific labeling of mitochondria over avidin or streptavidin. J. Histochem. Cytochem..

