# Supplementary material for: Cell Surface Proteomics Reveals Hypoxia-Regulated Pathways in Cervical and Bladder Cancer
Source: Proteomes. 2025 Aug 5;13(3):36. doi: 10.3390/proteomes13030036 (PMC12371898; doi:10.3390/proteomes13030036)
Supplement: Supplementary file 1 [file proteomes-13-00036-s001.zip › proteomes-3703616-supplementary.pdf]

proteomes MDPI

Table S1: GO enrichment terms and associated genes identified in SiHa cervical cancer cells.

**Term description**	**method**	**GO**	**Obs. proteins**	**Proteins list**
Mitochondrial matrix	Biotin-enriched	CC	35	ATP5F1D,ERAL1,MRPL53,MRPS9,TBRG4,MRPS18B,LRPPRC,NUBPL,PTCD1,RPUSD4,MRPL38,MRPS31,FARS2,TRIT1,BCAT2,TMLHE,MRRF,MRPL24,DAP3,SDHAF4,MRPS2,MRPL14,MRPS16,MRPS15,SUOX,VDAC1,MRPS12,FASTKD2,SDHAF3,MRPL22,MMAB,MRPL37,MRM1,SUCLA2,LARS2
Mitochondrial membrane			39	ATP5F1D,THG1L,ERAL1,MRPL53,MRPS9,MRPS18B,GPAM,SFXN5,MTERF3,HK2,SLC25A32,MRPL38,BOK,COQ8B,MRPS31,TMEM186,NDUFA4,MIGA2,TMEM126B,MRPL24,MARC1,DAP3,BNIP3,OMA1,MRPS2,MRPL14,MRPS16,MRPS15,NDUFA8,BPHL,VDAC1,MRPS12,CHCHD3,PISD,TAMM41,MRPL22,TIMM23,MRPL37,SLC25A16
Mitochondrial gene expression		BP	20	MRPL53,MRPS9,TBRG4,MRPS18B,PTCD1,RPUSD4,MRPL38,MRPS31,TRIT1,MRPL24,DAP3,MRPS2,MRPL14,MRPS16,MRPS15,MRPS12,FASTKD2,MRPL22,MRPL37,LARS2
Mitochondrial matrix	whole-cell lysate	CC	114	NDUFAF7,MRPS10,MRPS35,MPG,MRPL32,MRPL27,MRPL51,MRPS7,MRPL34,PTCD3,COQ3,ERAL1,MRPL53,MRPS9,TBRG4,MRPS18B,LRPPRC,TRMT5,MECR,DGUOK,FASTKD3,MRPL3,MCCC1,MRPS5,MMUT,NUBPL,GUF1,MARS2,MRPS17,COQ5,MRPL17,MRPL10,PTCD1,MRPL33,MRPL16,MRPL58,SDHAF2,TRMT61B,CBR4,MRPL39,MRPL13,MRPL4,MRPL38,MRPL48,MRPL11,PDP2,MRPL57,EXD2,CA5B,D2HGDH,MRPS31,FARS2,MRPS24,MRPS23,YARS2,TOP3A,GADD45GIP1,MRPL54,MRPL40,MRPL35,MRPL20,PYCR2,AURKAIP1,MCCC2,DHRS2,MRPL24,MRPL21,TFB2M,IBA57,MRPL18,DAP3,RARS2,SDHAF4,MTG2,PARS2,MRPL41,MRPL14,MRPS18A,MRPL50,ISCA1,PCCA,ATAD3A,FASTKD5,RPUSD3,MRPL2,FDX2,MRPL19,MTHFD2,VDAC1,MRPL23,PYCR1,MRPS12,FASTKD1,PNPT1,MRPL55,SDHAF3,MRPL47,MRPS22,MALSU1,NSUN4,TFAM,MRPS14,PDK2,MRPS30,MRPS27,DGLUCY,MRPL42

				,EARS2,MRPS21,POLRMT,MRPL37,MTERF4,SUCLA2,MRPL28
Mitochondrial membrane			103	MRPS10,MRPS35,GDAP1,MRPL32,RPS6KB1,MRPL27,MRPL51,MRPS7,MRPL34,TIMM10B,PTCD3,COQ3,ERAL1,PNPLA8,MRPL53,MRPS9,MRPS18B,MRPL3,GPAM,CPT1A,MTFP1,TIMM29,ECSIT,SLC27A3,MRPS5,GUF1,MRPS17,EXOG,COQ5,MRPL17,MRPL10,HK2,MRPL33,MRPL16,MRPL58,MRPL39,MRPL13,MRPL4,MRPL38,MRPL48,MRPL11,MRPL57,EXD2,BOK,COQ8B,MRPS31,MRPS24,TMEM11,MRPS23,TIMM22,GADD45GIP1,PTPMT1,MRPL54,MRPL40,MRPL35,NDUFA4,MRPL20,C15orf48,AURKAIP1,CASP8,MRPL24,MRPL21,MT-ATP8,MARC1,MARC2,MRPL18,DAP3,BNIP3,MTG2,OMA1,MRPL41,MRPL14,MRPS18A,MRPL50,UBIAD1,DNAJC11,ATAD3A,BNIP3L,MRPL2,COX16,MRPL19,MCUB,VDAC1,GATM,MRPL23,MRPS12,TAMM41,MRPL55,MRPL47,MRPS22,MFN1,MRPS14,MRPS30,MRPS27,TOMM5,MRPL42,PLSCR3,MRPS21,TIMM23,MRPL37,AKAP1,MRPL28,ATAD3B
Mitochondrial gene expression		BP	76	MRPS10,MRPS35,MRPL32,MRPL27,MRPL51,MRPS7,MRPL34,PTCD3,MRPL53,MRPS9,TBRG4,MRPS18B,TRMT5,GATB,FASTKD3,MRPL3,MRPS5,MRPS17,MRPL17,MRPL10,PTCD1,MRPL33,MRPL16,MRPL58,TRMT61B,MRPL39,MRPL13,MRPL4,MRPL38,MRPL48,MRPL11,MRPL57,MRPS31,MRPS24,MRPS23,YARS2,GADD45GIP1,MRPL54,MRPL40,MRPL35,MRPL20,AURKAIP1,MRPL24,MRPL21,TFB2M,MRPL18,DAP3,QRSL1,RARS2,MRPL41,MRPL14,MRPS18A,MRPL50,FASTKD5,MRPL2,MRPL19,MRPL23,MRPS12,FASTKD1,PNPT1,MTO1,MRPL55,MRPL47,MRPS22,TFAM,MRPS14,MRPS30,MRPS27,GATC,MRPL42,EARS2,MRPS21,POLRMT,MRPL37,MTERF4,MRPL28

Abbreviation: CC, Cellular Component; BP, Biological Process; GO, Gene Ontology; Obs., Observed

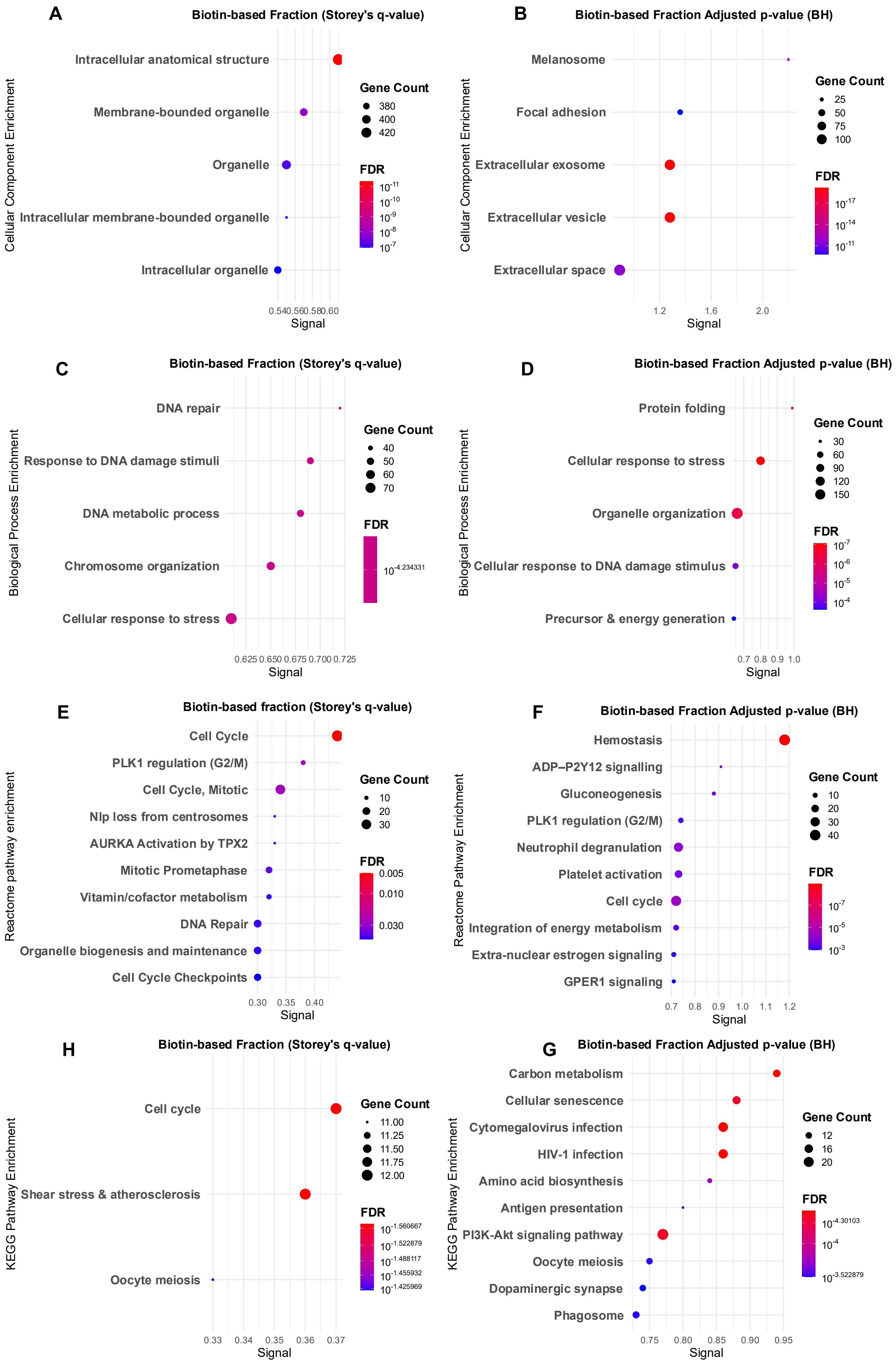
A
Biotin-based Fraction (Storey's q-value)
Cellular Component Enrichment
Intracellular anatomical structure
Membrane-bounded organelle
Organelle
Intracellular membrane-bounded organelle
Intracellular organelle
0.54 0.56 0.58 0.60
Signal
Gene Count
380
400
420
FDR
10^{-11}
10^{-10}
10^{-9}
10^{-8}
10^{-7}
B
Biotin-based Fraction Adjusted p-value (BH)
Cellular Component Enrichment
Melanosome
Focal adhesion
Extracellular exosome
Extracellular vesicle
Extracellular space
1.2
1.6
2.0
Signal
Gene Count
25
50
75
100
FDR
10^{-17}
10^{-14}
10^{-11}
C
Biotin-based Fraction (Storey's q-value)
Biological Process Enrichment
DNA repair
Response to DNA damage stimuli
DNA metabolic process
Chromosome organization
Cellular response to stress
0.625 0.650 0.675 0.700 0.725
Signal
Gene Count
40
50
60
70
FDR
$10^{-4.234331}$
D
Biotin-based Fraction Adjusted p-value (BH)
Biological Process Enrichment
Protein folding
Cellular response to stress
Organelle organization
Cellular response to DNA damage stimulus
Precursor & energy generation
0.7 0.8 0.9 1.0
Signal
Gene Count
30
60
90
120
150
FDR
10^{-7}
10^{-6}
10^{-5}
10^{-4}
E
Biotin-based fraction (Storey's q-value)
Reactome pathway enrichment
Cell Cycle
PLK1 regulation (G2/M)
Cell Cycle, Mitotic
Nlp loss from centrosomes
AURKA Activation by TPX2
Mitotic Prometaphase
Vitamin/cofactor metabolism
DNA Repair
Organelle biogenesis and maintenance
Cell Cycle Checkpoints
0.30 0.35 0.40
Signal
Gene Count
10
20
30
FDR
0.005
0.010
0.030
F
Biotin-based Fraction Adjusted p-value (BH)
Reactome Pathway Enrichment
Hemostasis
ADP–P2Y12 signalling
Gluconeogenesis
PLK1 regulation (G2/M)
Neutrophil degranulation
Platelet activation
Cell cycle
Integration of energy metabolism
Extra-nuclear estrogen signaling
GPER1 signaling
0.7 0.8 0.9 1.0 1.1 1.2
Signal
Gene Count
10
20
30
40
FDR
10^{-7}
10^{-5}
10^{-3}
H
Biotin-based Fraction (Storey's q-value)
KEGG Pathway Enrichment
Cell cycle
Shear stress & atherosclerosis
Oocyte meiosis
0.33 0.34 0.35 0.36 0.37
Signal
Gene Count
11.00
11.25
11.50
11.75
12.00
FDR
$10^{-1.560667}$
$10^{-1.522879}$
$10^{-1.488117}$
$10^{-1.455932}$
$10^{-1.425969}$
G
Biotin-based Fraction Adjusted p-value (BH)
KEGG Pathway Enrichment
Carbon metabolism
Cellular senescence
Cytomegalovirus infection
HIV-1 infection
Amino acid biosynthesis
Antigen presentation
PI3K-Akt signaling pathway
Oocyte meiosis
Dopaminergic synapse
Phagosome
0.75 0.80 0.85 0.90 0.95
Signal
Gene Count
12
16
20
FDR
$10^{-4.30103}$
10^{-4}
$10^{-3.522879}$

Figure S1. Comparison of Differential Abundant Proteins Results in UMUC3 Cells Using Storey's q-value vs Benjamini–Hochberg (BH) Adjusted p-values UMUC3 bladder cancer cells were cultured under normoxia (21% O_2) and hypoxia (0.1% O_2) for 48 hours. Differentially abundant proteins in the biotin-enriched fraction were identified based on a fold-change threshold ($|log_2FC| \geq 1$), using two significance criteria: (A) unadjusted raw p-value < 0.05 and (B) BH-adjusted p-value < 0.05. The overlap and differences between the two filtering approaches are shown, illustrating the trade-off between statistical stringency and detection sensitivity in a large dataset (n = 24). This comparison supports the use of BH adjustment in the main analysis while providing transparency regarding proteins retained or excluded under each method.
